# Whole-genome analysis of *Ligilactobacillus salivarius* L33, a potential probiotic strain isolated from chicken gastrointestinal tract

**DOI:** 10.1128/spectrum.01591-24

**Published:** 2025-08-15

**Authors:** Seyedeh Arezoo Maniee, Sahar Mahmoodian, Javad Zamani Amirzakaria, Amir Meimandipour, Vahid Shariati, Elahe Tavakol

**Affiliations:** 1 Department of Cellular and Molecular Biology, College of Science and Biology Technologies, University of Science and Culture 577243, Tehran, Iran; 2 Department of Animal Biotechnology, National Institute of Genetic Engineering and Biotechnology, Tehran, Iran; 3 Plant Molecular Biotechnology Department, National Institute of Genetic Engineering and Biotechnology, Tehran, Iran; 4 Department of Plant Genetics and Production, University of Shiraz 37551, Shiraz, Iran; Università degli Studi di Napoli Federico II, Naples, Italy

**Keywords:** *Ligilactobacillus salivarius*, whole-genome analysis, potentially probiotic strain, broiler chicken

## Abstract

*Ligilactobacillus salivarius* is a widely used probiotic in human and animal nutrition, particularly for enhancing performance in poultry farming. Supplementing feed with this bacterium has shown benefits such as reducing *Salmonella Enteritidis* colonization and promoting weight gain. This study investigated the probiotic potential of a newly isolated strain, *L. salivarius* L33, obtained from the gut of broiler chickens. We employed a series of *in vitro* tests to assess its probiotic characteristics. These tests evaluated its resistance to simulated digestive conditions, lack of hemolytic activity on blood cells, adherence ability to intestinal surfaces, antibiotic susceptibility, and antimicrobial activity. Whole-genome sequencing of *L. salivarius* L33 using the Illumina NovaSeq6000 platform revealed 1,870 genes encoding 1,797 proteins. Comparative analysis identified 111 unique genes involved in critical cellular functions such as replication, repair, and defense mechanisms. Additionally, the genome harbored a bacteriocin gene encoding salivaricin, a potent antimicrobial compound. Furthermore, genes associated with key probiotic properties were identified, including stress tolerance, adhesion to gut epithelia, and survival in acidic and bile salt environments. These findings strongly suggest the probiotic potential of *L. salivarius* L33. The presence of genes linked to established probiotic functionalities and its similarity to known probiotic strains of *L. salivarius* make it a promising candidate for further research and development as a novel probiotic for poultry applications.

**IMPORTANCE:**

*Ligilactobacillus salivarius* is a well-studied probiotic with proven benefits in poultry farming, including improved growth and reduced pathogen colonization. This study characterizes a novel strain, *L. salivarius* L33, isolated from broiler chickens, and demonstrates its potential as a probiotic through genomic and functional analyses. The strain’s genome reveals genes linked to stress tolerance, gut adhesion, and antimicrobial activity, including salivaricin, a bacteriocin with broad anti-pathogen effects. These findings are significant because they highlight L33’s promise as a natural, safe alternative to antibiotics in poultry production, aligning with global efforts to reduce antimicrobial resistance. Further development of this strain could enhance animal health, productivity, and food safety, offering economic and public health benefits.

## INTRODUCTION

Probiotics are defined as live microorganisms that, when administered in adequate amounts, confer a health benefit on the host (1). These beneficial microbes are particularly important in poultry, where they contribute significantly to enhancing animal health and productivity. Commonly added to poultry feed, they enhance animal well-being through various mechanisms (2). The poultry gut harbors a vast number of microorganisms, known as microbiota, with a bacterial population estimated between 10^10^ and 10^11^ CFU/g (3). Probiotics serve multiple roles in the poultry industry. They are used as dietary supplements, feed additives, and even antibiotic replacements (3, 4). Their versatility allows for easy combination with other additives to improve animal health and production (5). The established effectiveness of probiotics in combating bacterial diseases and zoonoses has strengthened the concept of “gut-derived immunity” in poultry (6).

*Lactobacillus salivarius* is a widely used probiotic in human and animal nutrition (7). In poultry specifically, it has been used for over 15 years to improve performance and enhance resistance to enteric infections (8). Studies suggest that *L. salivarius* reduces *Salmonella enteritidis* colonization through competitive exclusion (9). Shokryazdan et al. (10) evaluated a *Lactobacillus* mixture containing three *L. salivarius* strains isolated from chicken intestines (10). Their findings suggest improved body weight, weight gain, and feed conversion ratio at specific dietary concentrations. Additionally, the mixture lowered cholesterol and triglycerides while increasing beneficial bacteria and decreasing harmful ones, along with their enzymes such as β-glucosidase and β-glucuronidase. However, recent studies have highlighted that the functions of probiotics are strongly influenced by their specific strains, and as such, their biological effects should be assessed individually (11, 12). For newly discovered probiotics, it is essential to analyze and evaluate their probiotic function at the gene level to uncover additional biological functions and information (11).

Genomics has revolutionized how we identify and classify novel probiotics. Next-generation sequencing platforms used by various studies facilitate more precise taxonomic and functional characterization of new isolates. Multiple studies have examined the genes associated with the probiotic functionalities of *L. salivarius*. A recent study by Karlyshev and Gould (13) investigating the complete genome of *L. salivarius* strain 2102-15, isolated from the vaginal microbiome of a healthy woman, revealed the presence of diverse bacteriocin and immunity protein-encoding genes (13). These genes were identified on both the chromosome and one of the plasmids harbored by the bacteria. This finding suggests that *L. salivarius* 2102-15 possesses the genetic potential to produce compounds similar to salivaricin, nisin, and enterolysins, potentially contributing to its role in maintaining vaginal health. In another study by Jiang et al. (14) on the assessment of the safety and probiotic characteristics of *L. salivarius* CGMCC20700, whole-genome sequencing results showed that *L. salivarius* CGMCC20700 has a single scaffold of 1,737,577 bp, with an average guanine-to-cytosine (GC) ratio of 33.51% and 1,757 protein-coding genes (14). Sequences related to risk assessment, such as antibiotic resistance and virulence genes, further confirmed the strain as safe according to the results of antibiotic resistance, hemolytic, and acute oral toxicology tests. In the mentioned study, two gene clusters of antibacterial compounds and broad-spectrum antimicrobial activity were identified using genome mining tools and antibacterial spectrum tests.

In this study, whole-genome sequencing was employed to investigate the probiotic characteristics of a novel *L. salivarius* L33 strain isolated from the gastrointestinal tract of broiler chickens. Following genome sequencing, annotation and comparative analysis with other *L. salivarius* strains were performed to identify strain-specific genes. The study specifically focused on genes involved in bacteriocin biosynthesis, adhesion proteins, and stress resistance. Additionally, eggNOG-mapper, Kyoto Encyclopedia of Genes and Genomes (KEGG) pathway, and carbohydrate-active enzyme (CAZyme) analysis were used to assess the metabolic capabilities of the genome. This research provides valuable insights into the probiotic properties of this commensal *L. salivarius*, highlighting its potential as a novel probiotic for poultry applications.

## MATERIALS AND METHODS

### Sample collection and bacterial strain isolation

Briefly, ileal segments (5 cm from the distal end) were aseptically collected from 20 healthy, 4-week-old broiler chickens (Arian breed). To isolate lumen-associated bacteria, the ileal contents were rinsed three times with phosphate-buffered saline (PBS) and homogenized using a Bullet Blender (Next Advance, Inc., Troy, NY, USA). The resulting supernatant was subjected to serial dilution and plated on Man, Rogosa, and Sharpe (MRS; Ibresco Co., Iran) agar plates. These plates were incubated at 37°C under microaerophilic conditions (5% O_2_, 10% CO_2_, and balanced N_2_) for 16 hours. Sixty bacterial isolates were selected based on colony morphology, purified, and preserved at −80°C in MRS broth supplemented with 20% glycerol for subsequent phenotypic characterization and probiotic screening, as outlined below. Following the screening process, potential probiotic strains were identified through 16S rRNA gene sequencing, with sequence analysis performed using the nucleotide BLAST tool available through the National Center for Biotechnology Information.

### Tolerance to gastrointestinal tract conditions

Bacterial tolerance to the harsh environment of the gastrointestinal tract was evaluated using a modified method based on Farid et al. (15). Briefly, bacteria were cultured in MRS broth at 37°C for 18 hours. Following incubation, cells were harvested by centrifugation and washed with phosphate-buffered saline (pH 7.0). A 0.5 McFarland standard suspension was prepared and mixed with MRS broth (pH 3.0) at 10% (vol/vol) concentration. This suspension was then incubated at 37°C for 3 hours. Samples were collected at 0 and 3 hours with three replicates for each time point. Bacterial viability was assessed by colony-forming unit (CFU) enumeration. To assess bile salt resistance, a 0.5 McFarland standard suspension of bacteria was resuspended at 10% (vol/vol) in MRS broth supplemented with 0.3% (wt/vol) bile salts (Sigma, Germany). This suspension was incubated at 37°C for 8 hours. Samples were collected at 0 and 8 hours with three replicates for each time point. Bacterial growth was monitored by measuring the optical density (OD) at 600 nm wavelength.

### Hemolytic activity

The bacterial strain was cultured overnight in MRS broth medium at 37°C for approximately 18 hours. Following incubation, the culture was streaked onto a blood agar plate containing 5% (vol/vol) defibrinated sheep blood. The inoculated plate was then incubated at 37°C for 48 hours to assess hemolytic activity. To ensure experiment validity, a positive control strain, *Staphylococcus aureus* ATCC 25923, known for β-hemolysis on blood agar, was included alongside the test strain (16). Both the treatment and control samples were analyzed in triplicate to ensure the reliability of the results.

### Antimicrobial ability

The antibacterial properties of *L. salivarius* were evaluated using a broth microdilution method with standardized indicator bacteria. Five strains were chosen, representing both gram-positive (*Staphylococcus aureus* ATCC 25923 and *Listeria monocytogenes* ATCC 119115) and gram-negative (*Escherichia coli* ATCC 25922, *Salmonella enterica* subsp. *enterica* serovar Typhimurium ATCC 14028, and *Pseudomonas aeruginosa* ATCC 27853) bacteria. Overnight cultures of each indicator strain were prepared in nutrient broth (Ibresco, Iran) at 37°C. The cultures were then adjusted to a standardized inoculum density of approximately 1 × 10^8^ CFU/mL using a 0.5 McFarland turbidity standard. The standardized indicator bacterial cultures were then spread evenly onto the nutrient agar (Ibresco, Iran) surface, followed by storing at 4°C for 1 hour and then creating 6 mm diameter wells on plates using sterile Oxford cups. Each well was filled with 50 µL of overnight *L. salivarius* culture supernatant, and the plates were incubated aerobically at 37°C for 48 hours. After incubation, the plates were observed for the presence of inhibition zones surrounding the wells. The diameter of each inhibition zone was measured in millimeters. Each test was performed in triplicate to ensure data consistency and reliability.

### Antibiotic susceptibility

The susceptibility of the bacteria to various antibiotics was determined using the Kirby-Bauer disk diffusion method. A 0.5 McFarland standard turbidity suspension of the test organism was prepared in sterile broth and inoculated onto Mueller-Hinton agar plates using a sterile swab to achieve a confluent lawn. Commercially available antibiotic discs (Padtan Teb Company) containing specific concentrations of different antibiotics were then placed aseptically onto the inoculated agar surface using sterile forceps. The plates were incubated at 37°C for 18–24 hours to allow for bacterial growth and diffusion of antibiotics. Following incubation, the diameters of the zones of inhibition (clear zones with no bacterial growth) surrounding each antibiotic disc were measured using a ruler. The results were interpreted using established criteria from the Clinical and Laboratory Standards Institute (CLSI) guidelines, which categorize the bacteria as susceptible, intermediate, or resistant to the tested antibiotics.

### Adhesion to Caco-2 cells

The human colon carcinoma cell line, Caco-2, obtained from the National Institute of Genetic Engineering and Biotechnology, was used for the adhesion assay. Cells were cultured in high-glucose Dulbecco’s Modified Eagle’s Medium (DMEM) supplemented with 10% fetal bovine serum and 1% penicillin-streptomycin solution in a humidified incubator at 37°C with 5% CO_2_ (DNA biotech Co., Iran). The culture medium was replaced every other day until the cells reached approximately 80% confluency. The strain of interest (*L. salivarius* L33) was cultured in MRS broth medium, and its absorbance was adjusted to a 0.5 McFarland standard using fresh DMEM. This standardization ensures a consistent inoculum density for the adhesion assay. Following trypsinization and cell counting, Caco-2 cells (4 × 10^5^ cells/mL) were seeded onto a 24-well plate. Each well received 100 µL of the standardized bacterial suspension, and the plate was incubated at 37°C for 2 hours to allow for bacterial adhesion. After incubation, the cells were washed twice with PBS to remove non-adherent bacteria. Subsequently, the cells were fixed with methanol for further processing. Crystal violet staining was employed for 5 minutes to visualize the adherent bacteria. *Escherichia coli* strain ATCC 25922, a non-pathogenic strain, and *Lactobacillus plantarum* ATCC 14917, a well-established probiotic strain with documented adhesion, were included as controls in the adhesion assay to compare the adherence potential of *L. salivarius* L33. The methodology for this assay was adapted from the protocol described in reference 17.

### Sample preparation and DNA extraction

The bacterial strain was cultured in MRS broth (Ibresco, Iran) at 37°C for 16–18 hours. Following incubation, the bacterial cells were harvested by centrifugation at 8,000 × *g* for 10 minutes using a centrifuge (Heraeus, Germany). Total genomic DNA was extracted from the cell pellet using a commercially available DNA extraction kit (Pouya Gene Azma Co., Iran) according to the manufacturer’s instructions. The purity and quantity of the extracted DNA were evaluated spectrophotometrically at a wavelength of 260 nm using a NanoDrop ND-1000 UV-Vis Spectrophotometer (Thermo Fisher Scientific, USA).

### Whole-genome sequencing

The Illumina NovaSeq6000 platform was used to sequence the genomic DNA of bacteria (2 × 150 paired ends). The sequencing resulted in 4,247,834 paired-end reads. We assessed the read quality with FASTQC (version 0.11.9) and removed low-quality reads using Trimmomatic (version 3.39). *De novo* assembly was done with SPAdes (version 3.15.1) (18). Genome annotation was carried out locally, using PROKKA (version 1.11). EggNOG-mapper version 2 tool (19) was applied for functional classification of proteins. Blast KOALA (version 3.0) was used for Kyoto Encyclopedia of Genes and Genomes mapping of the predicted genes. Carbohydrate-active enzymes were searched against the CAZy database. MOB-suit standalone tool (20) was used to predict the plasmid sequences from the *de novo* assembled contigs. Clustered regularly interspaced palindromic repeats (CRISPR) inside the assembly were evaluated using CRISPR Detect (version 2.4) (21). PHAge Search Tool Enhanced Release (22) was utilized for the identification and annotation of putative prophage sequences inside the bacterial assembly. Visualization of the genome assembly was performed by the Artemis tool (version 18.1.0) (23), while its metrics were calculated with the Quality Assessment Tool (version 5.2.0).

### Evaluation of probiotic features

The existence of antibiotic resistance genes was evaluated using the Resistance Gene Identifier. The BLAST tool was used for identifying different probiotic-endowing features in the genome.

### Pan-genome analysis

This study employed a pan-genome analysis to investigate the complete genetic makeup of the *L. salivarius* L33 draft genome and its related strains. Roary, a bioinformatics software specifically designed for pan-genome analysis, was utilized for this purpose. The first step involved obtaining the bacterial genome sequences in FASTA format from a public genome database. Subsequently, these sequences were processed using PROKKA to generate GFF3 files that are compatible with Roary. The generated GFF3 files were then imported into Roary for pan-genome analysis. Roary identifies core genes (present in all strains), soft core genes (present in most strains), and shell genes (variable across strains). This analysis provides insights into the genomic diversity and functional repertoire of the bacterial population under investigation. Furthermore, Roary facilitates the construction of a maximum-likelihood phylogenetic tree based on the core genome alignment. This phylogenetic tree helps visualize the evolutionary relationships between the L33 strain and its related genomes based on their core genes.

### Statistical analysis

Statistical analysis was conducted using SPSS version 19.0 (SPSS Inc., Chicago, IL, USA). A *t*-test was performed to compare the groups, and a *P*-value of less than 0.05 (*P* < 0.05) was considered statistically significant, indicating that the observed differences were unlikely to have occurred by chance.

## RESULTS

### *In vitro* probiotic properties of *L. salivarius* L33

**Fig 1:**
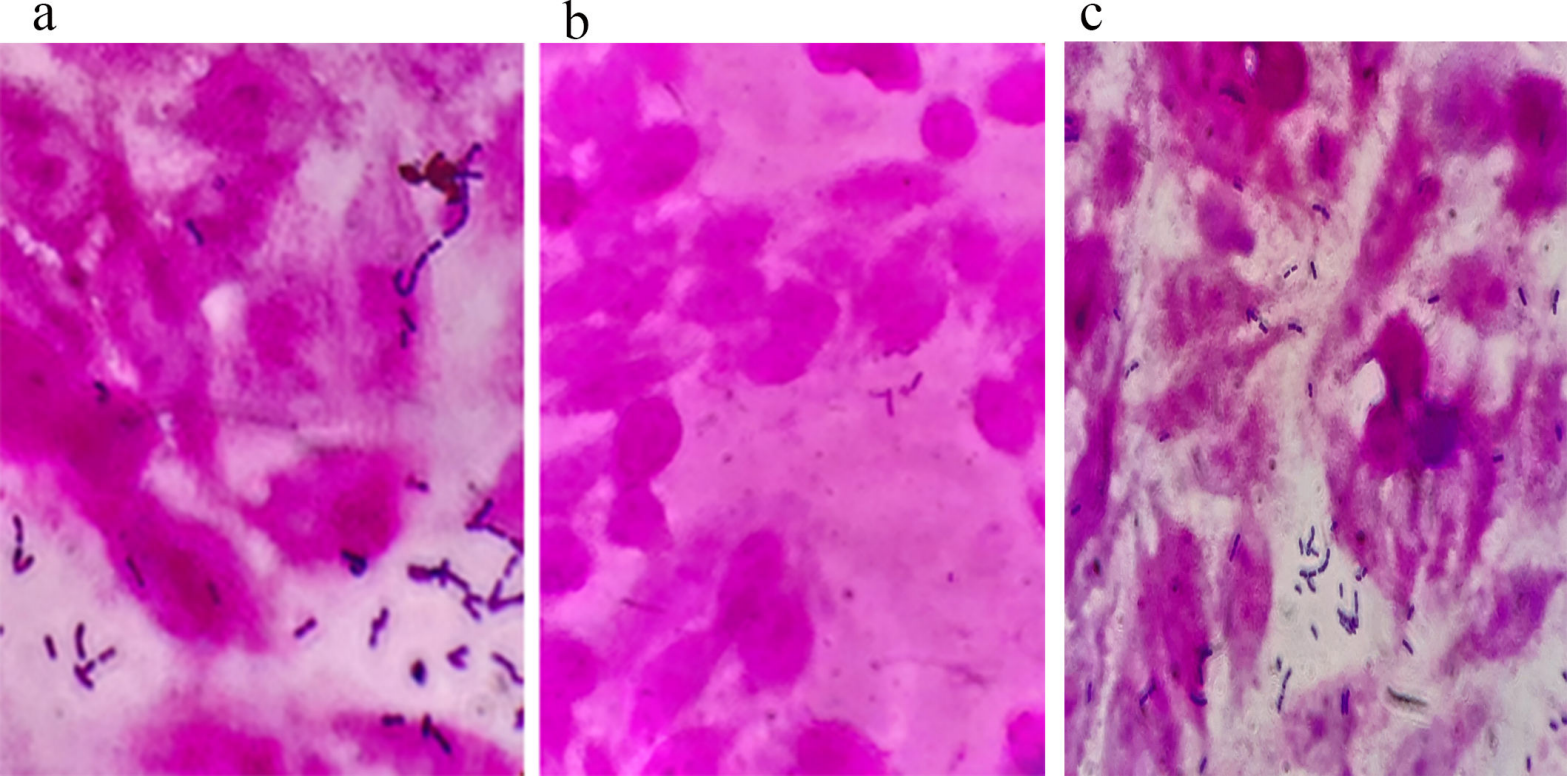
Microscopic analysis of *Lactobacillus salivarius* L33 binding to Caco-2 cells. (a) *L. salivarius* L33, (b) *E. coli* ATCC 25922, and (c) *L. plantarum* ATCC 14917.

Probiotic strains must possess the ability to survive and potentially multiply within the harsh environment of the gastrointestinal tract, which includes low pH due to gastric acid and the presence of bile salts (24). An *in vitro* evaluation of the strain’s capability to tolerate these conditions exhibited a survival rate of approximately 92.3% in simulated gastric juice pH 3 (*P* value > 0.05) and 65% in the presence of high bile salt concentration (*P* value < 0.05) (Tables 1 and 2). These results suggest the strain’s potential to survive passage through the gastrointestinal tract. Furthermore, the strain displayed no hemolytic activity on blood agar, indicating it does not lyse red blood cells. Adhesion of bacteria to intestinal epithelium is a main feature of probiotic bacteria. The adhesion is controlled by the composition, structure, and production of proteinaceous secretory adhesion (24). Microscopic analysis revealed that the *L. salivarius* L33 strain exhibited a binding affinity to Caco-2 cells that was significantly higher than *E. coli*, while demonstrating adhesion similar to that of *L. plantarum*, across various observed fields (Fig. 1). According to the antibiotic sensitivity test, *L. salivarius* L33 showed good antibiotic sensitivity (Table 3). Consistent with bioinformatics analysis, the bacteria showed intermediate sensitivity to tetracycline. The *in vitro* antagonistic effects of *L. salivarius* L33 were evaluated against a panel of indicator pathogens. The well diffusion method was employed to assess its ability to inhibit the growth of these pathogens. The strain exhibited antimicrobial activity against various pathogenic bacteria, including *E. coli* ATCC 25922, *S. enterica* serovar Typhimurium ATCC 14028, *S. aureus* ATCC 25923, *L. monocytogenes* ATCC 119115, and *P. aeruginosa* ATCC 27853 (Table 4). The observed inhibition zones were greater than 2 mm in diameter.

**TABLE 1:** Probiotic properties of *L. salivarius* L33 against acidic conditions^*a*^

Time (h)	Control			Treatment			*P* value
	pH	Log10 CFU/mL	Survival rate (%)	pH	Log10 CFU/mL	Survival rate (%)	
3	5.8	7.07	100	4	6.72	95.1	0.058
				3	6.5	92.3	0.052

^*a*^ The CFU/mL values for both treatment and control groups are averages of three values.

**TABLE 2:** The survival rate of *L. salivarius* L33 against bile salt^*a*^

Time (h)	OD_600_ control	Treatment		*P* value
		OD_600_	Survival rate (%)	
**8**	**2.92**	**1.9**	**65**	0.032*
0	**0.001**	**0.001**	**–^*b*^**	**–**

^*a*^ The OD_600_ values for both treatment and control groups are analyzed with t-test at 95% confidence level. Each number is the average of three replicates.

^*b*^ "–” denotes not applicable.

**TABLE 3:** Antibiotic activity assay of *L. salivarius* L33

Antibiotic	Sensitivity
Clindamycin	Susceptible
Tetracycline	Intermediate
Chloramphenicol	Susceptible
Penicillin	Susceptible
Erythromycin	Susceptible
Ampicillin	Susceptible
Rifampin	Susceptible
Tylosin	Susceptible
Florfenicol	Susceptible
Kanamycin	Resistant

**TABLE 4:** Antimicrobial ability of *L. salivarius* L33 in the presence of different pathogenic bacteria^*a*^

Strain	*S. aureus*	*Bacillus cereus*	*L. monocytogenes*	*S. enterica*	*E. coli*	*P* value
*L. salivarius* L33	>2	>2	>2	>2	>2	0.081
*L. reuteri* S8	>2	>2	>2	>2	>2	0.06

^*a*^ Each value represents the mean derived from triplicate measurements, with the dimensions expressed in millimeters.

### General genome features and taxonomic classification

**Fig 2:**
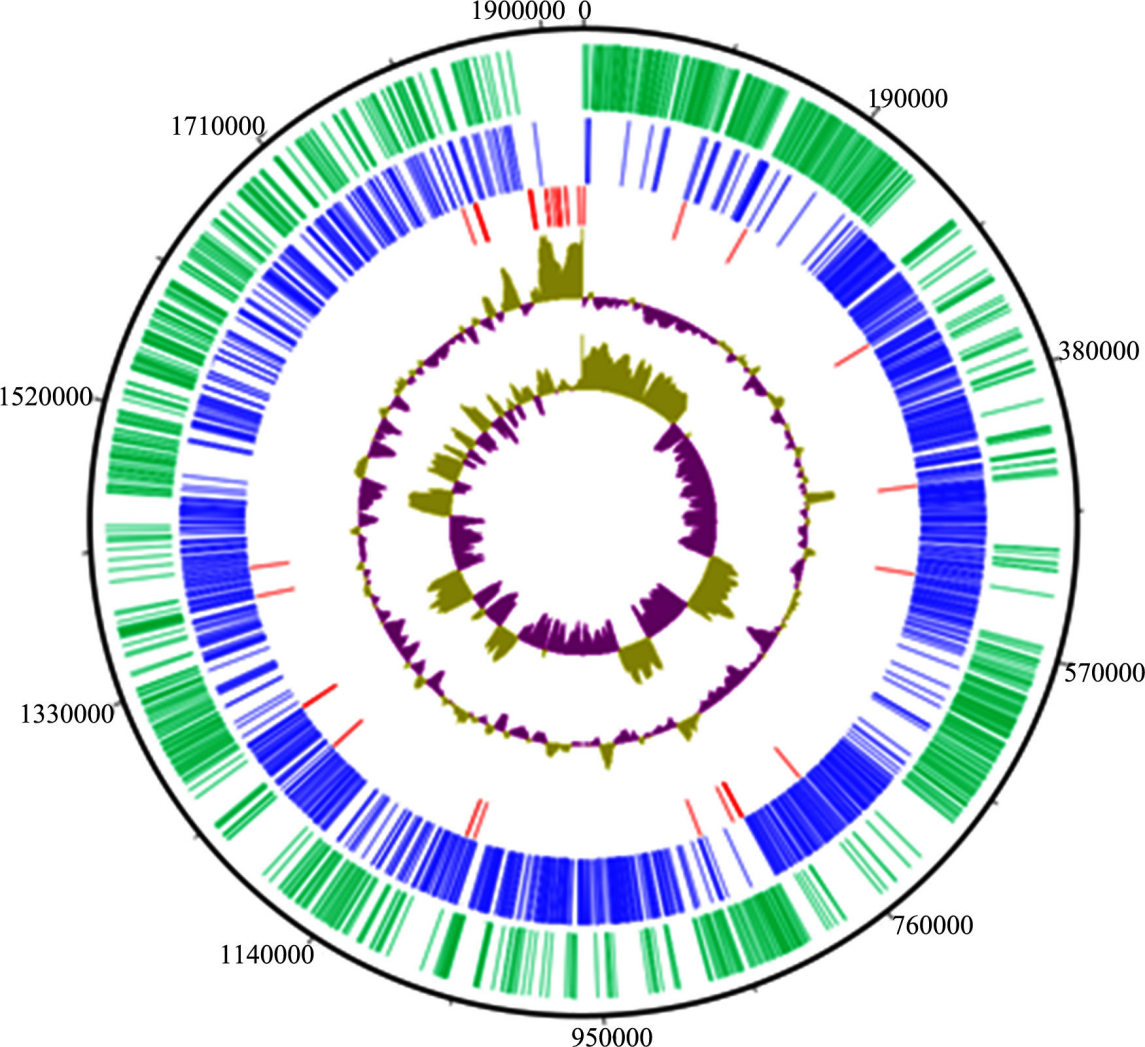
Circular genome map of *L. salivarius* L33 with seven circles. Marked information is displayed from the outer circle to the innermost as follows: genome size (black), CDSs on the forward strand (green), CDSs on the reverse strand (blue), rRNA and tRNA (red), and GC content and GC skew.

The whole genome of *L. salivarius* L33 was sequenced, and genomic features were investigated through comprehensive bioinformatics analysis. The estimated coverage of the sample was approximately 600×, indicating a high level of observation detail, allowing for a thorough analysis of the adhesion characteristics of the bacteria. According to the results, *de novo* assembly of the reads using SPAdes resulted in 372 contigs, with an *N*_50_ value of 70,157. Draft genome of *L. salivarius* L33 has a length of 1,925,881 bp, with a GC content of 32.62% (Table 5; Fig. 2). About 1,870 ORFs and 1,797 protein coding sequences with an average length of 907 bp were identified, and these occupied 86.01% of the genome. A total of 83% and 84% of CDSs were assigned to at least one protein family and COG categories, respectively. The genome contains 5 rRNA and 68 tRNA corresponding to all 20 natural amino acids. The plasmid contigs were successfully separated using the MOB-suite software, which facilitated the identification and assembly of three distinct plasmids. Based on the analysis, the sizes of the plasmids were estimated to be 7,646, 19,542, and 158,809 bp, respectively. These size estimates provide valuable insight into the genomic structure of the plasmids and their potential functional roles.

**TABLE 5:** General genome feature of *L. salivarius* L33

Genome size	1,896,929
GC content (%)	32.62
Total genes	1,870
Total protein	1,797
tRNA	66
rRNA	6

**Fig 3:**
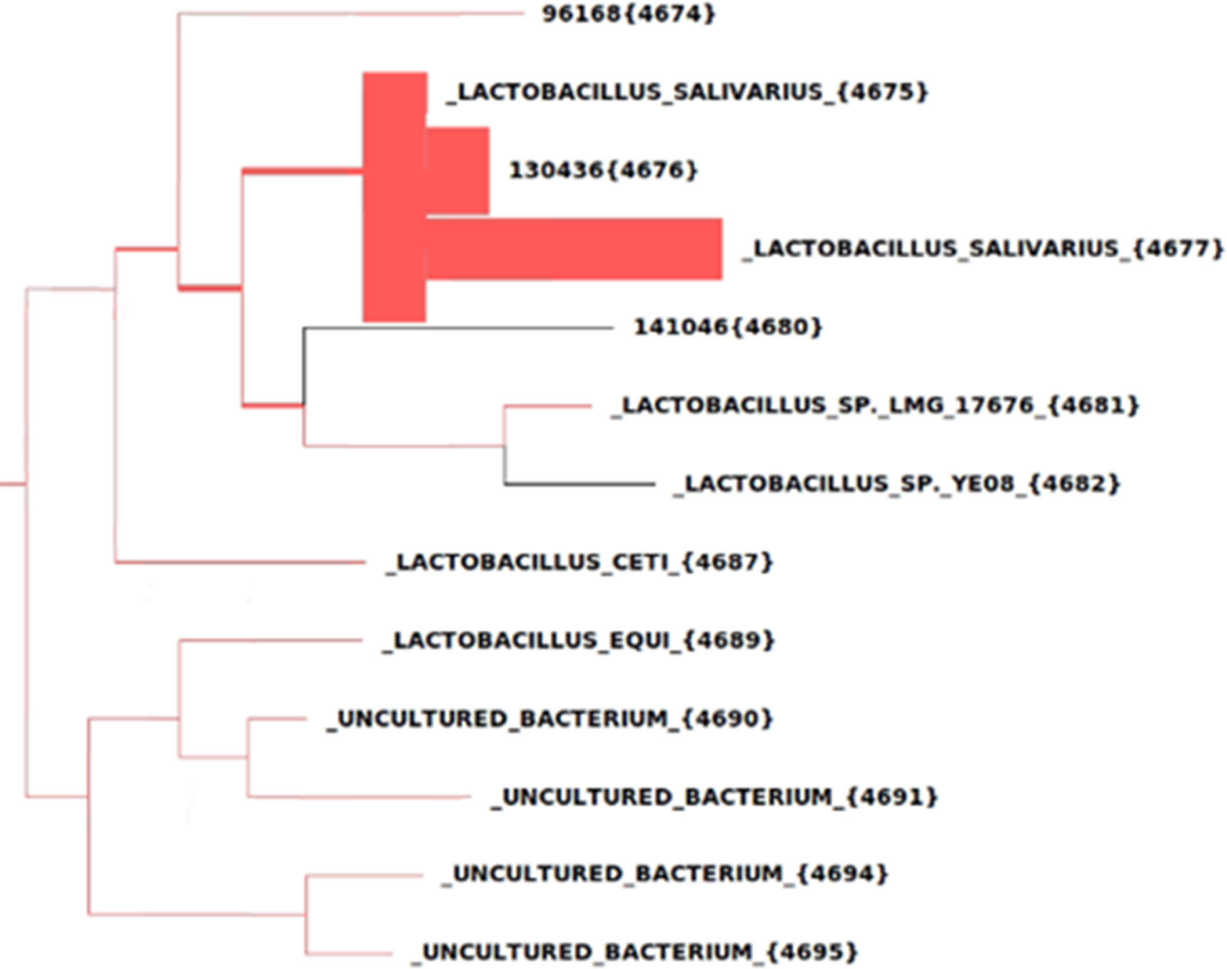
Phylogenetic model result from PhyloSift analysis for high-resolution identification of bacteria. The thickness of branches indicates the number of reads supporting each particular species.

Furthermore, one clustered regularly interspaced short palindromic repeat array on a contig, with a length of 51,698 (Table 6), and one intact prophage region on a contig, with a length of 424,006 bp, were predicted (Table 7). The taxonomic assignment of the genome was identified using the PhyloSift package. This tool aligns raw reads to 40 marker genes to make a weighted probability score for taxonomic classification (25). In other words, PhyloSift analysis generates a phylogenetic model and identifies organisms with high resolution using raw Illumina reads. The analysis identified the close relative of this genome at the species level and revealed that the L33 strain is closely related to *L. salivarius* (Fig. 3).

**TABLE 6:** Predicted CRISPR region on *L. salivarius* L33 genome

CRISPR start position	CRISPR end position	CRISPR length	Repeated sequence	No. of spacers
7591	9592	2,001	GTTTCAGAAGTATGTTAAATCAATAAGGTTAAGACC	29

**TABLE 7:** Predicted PHAGE region on *L. salivarius* L33 genome

Region	Region length	Completeness	Position	PHAGE_HIT_PROTEIN_NUM	PHAGE_SPECIES_NUM	GC percentage
1	48.1 kb	Intact	226975–275078	35	16	

### Pan-genome analysis of genomes

**Fig 4:**
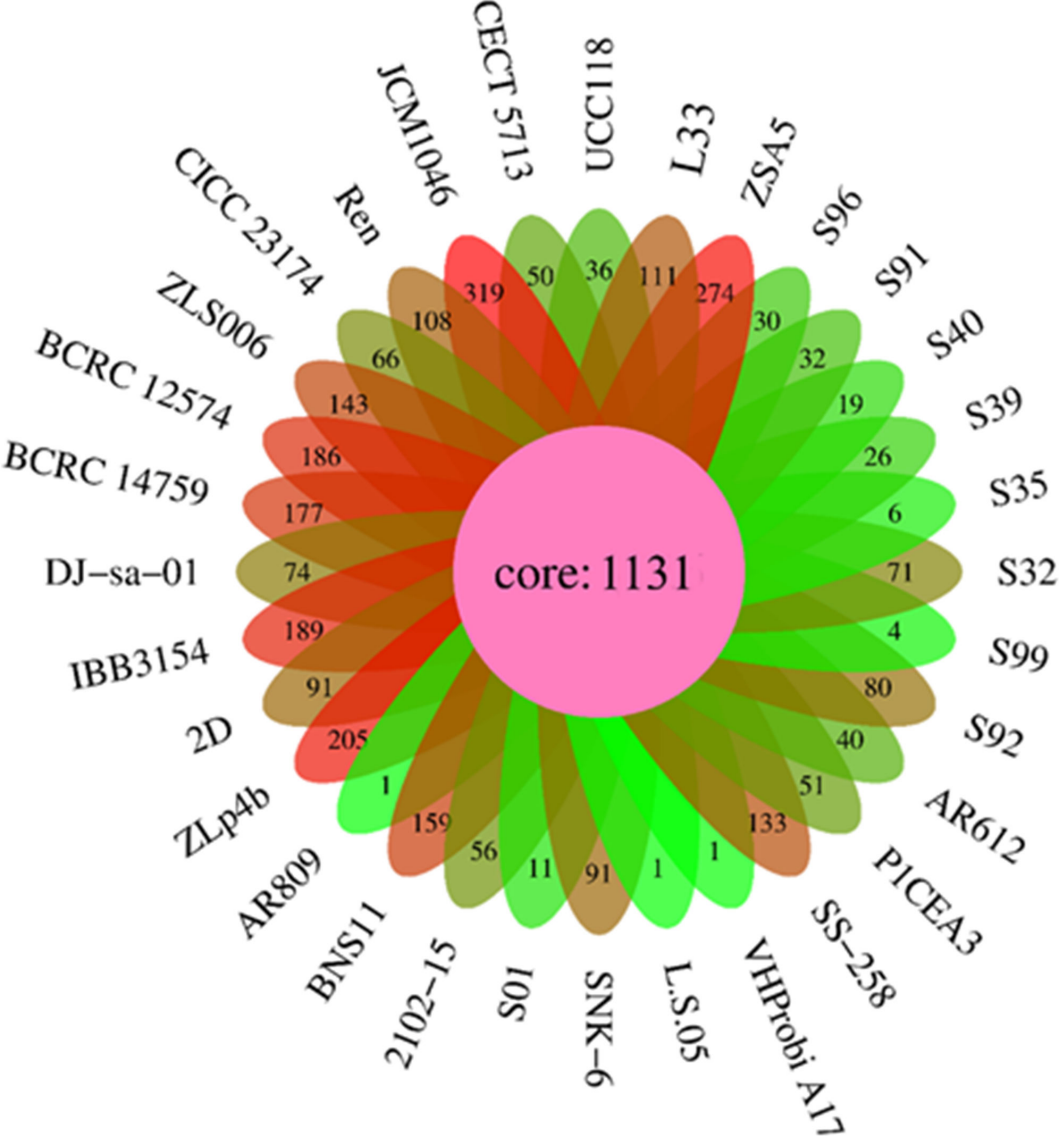
Venn diagram illustrating the number of orthologous gene clusters of core genes (center part) and the number of unique genes among 32 *L*. *salivarius* strains.

The pan-genome analysis is applied for evaluating genome diversity within a given species (26). The pan-genome encompasses the total set of genes from all strains, while the core genome is the total number of genes shared by all strains (26). The pan-genome of *L. salivarius* strains with available complete genomes and the L33 strain was constructed using Roary. The *L. salivarius* pan-genome genes clustered into core genes that were found in >95% of genomes, accessory genes, including shell genes found in 15%–95% of genomes, and cloud genes found in <15% of genomes. The analysis showed that the pan-genome of *L. salivarius* species (33 genomes) consists of 7,531 orthologous gene clusters (1,131 gene clusters as core, 1,585 gene clusters as shell, and 4,730 gene clusters as cloud) (Fig. 4). The number of strain-specific genes ranged from one gene found only in *L. salivarius* 05, VHProbi A17, and AR809 to 319 genes only found in JCM1046.

**Fig 5:**
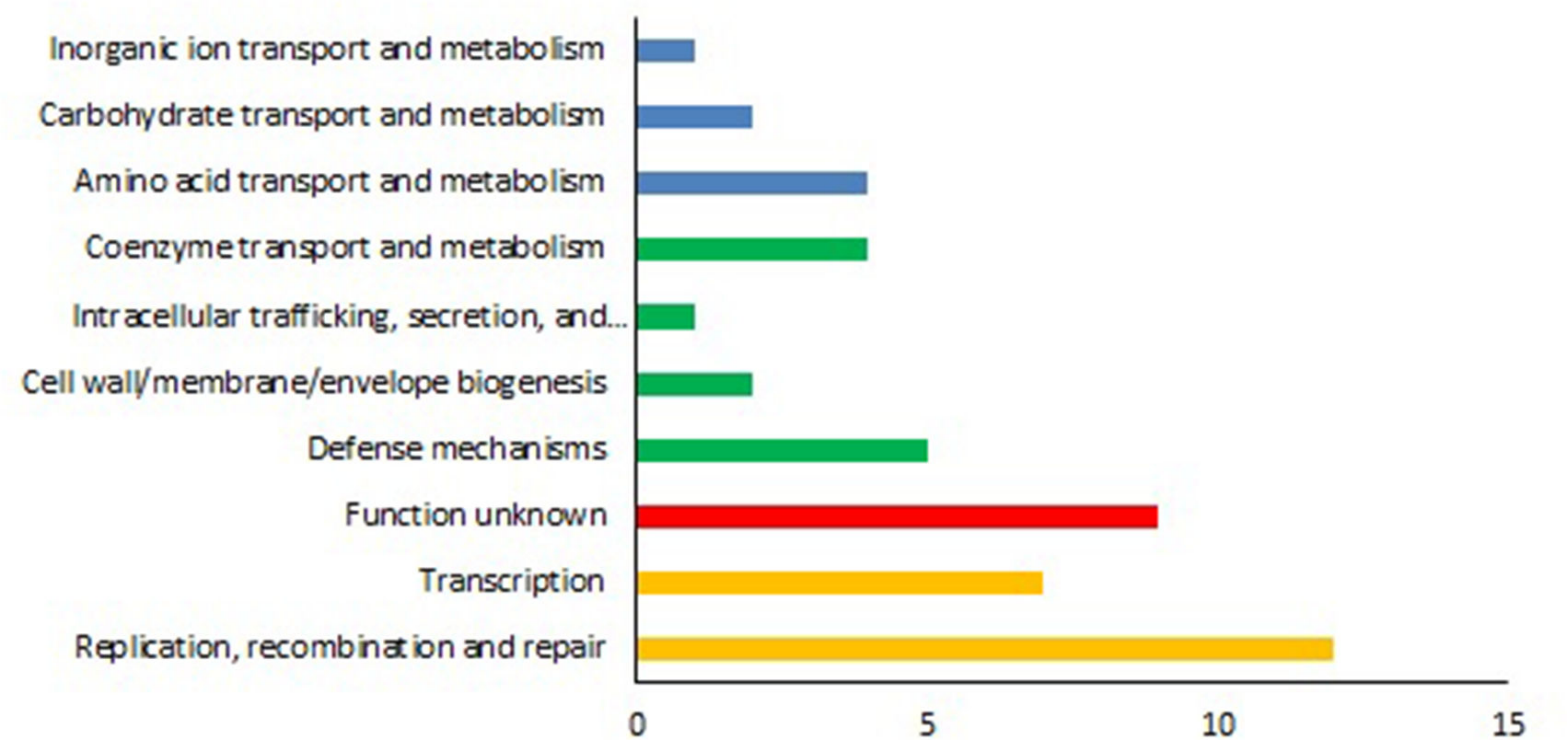
Classification of *L. salivarius* L33 unique genes assigned to COG categories using eggNOG-mapper.

**Fig 6:**
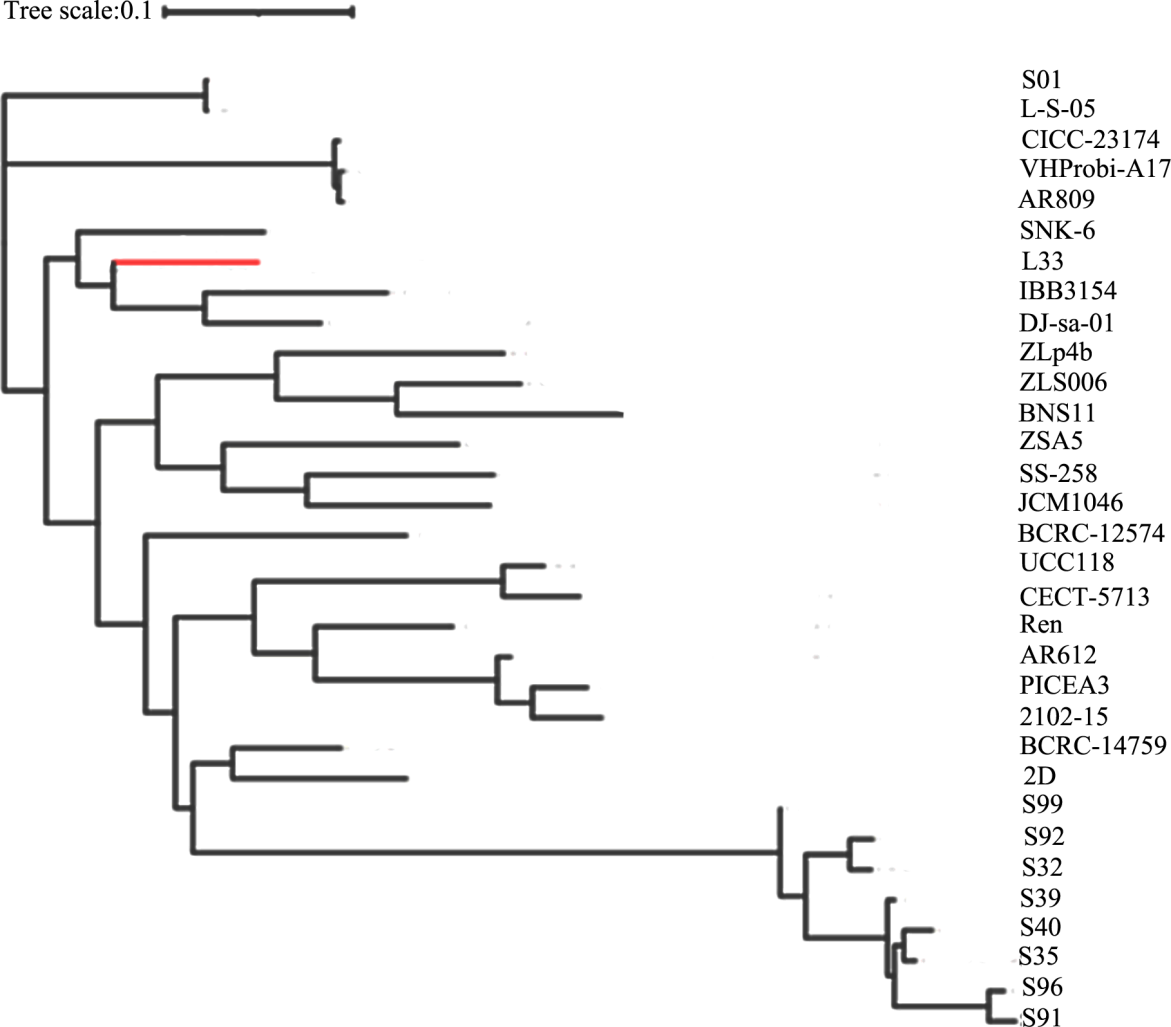
Phylogeny of *Lactobacillus* species based on a pan-genome Roary analysis of 32 strains with core gene sequences.

Furthermore, as shown in Fig. 4, L33 possesses 111 strain-specific genes. Of these, 28 genes originated from the prophage region. The COG classification of the proteins encoded by the remaining 83 genes was performed using EggNOG-mapper (Fig. 5). They were grouped into 11 functional categories. The majority (12 genes, Class L) were classified under replication, recombination, and repair. A total of seven genes were categorized as transcription (Class K), five genes as defense mechanisms (Class V), two genes as cell wall/membrane/envelope biogenesis (Class M), four genes as amino acid transport and metabolism (Class E), four genes as coenzyme transport and metabolism (Class H), two genes as carbohydrate transport and metabolism (Class G), and one gene as inorganic ion transport and metabolism (Class P). To investigate the phylogeny of *L. salivarius* strain L33, a phylogenetic tree was constructed based on the alignment of the core genes. As shown in Fig. 6, the closest evolutionary relatives of L33 are IBB3154, DJ-sa-01, and SNK6, all of which are poultry probiotics. *L. salivarius* IBB3154 is a probiotic strain that was isolated from chicken feces. Its different probiotic features have been well described (27). *L. salivarius* SNK6 is a probiotic strain whose beneficial effect in laying hens has been reported (28). According to the results, dietary supplementary *L*. *salivarius* SNK-6 in laying hens activated the intestinal mucosal immune system by regulating cecal microbial composition. *L. salivarius* DJ-sa-01 is also a potential probiotic strain, which has been isolated from the chicken small intestine (29).

### Functional classification

We evaluated the COG and KEGG profiles of *L. salivarius* using EggNOG-mapper and Blast-koala tools, respectively. Of the 1,797 CDSs, a total of 1,574 CDSs were assigned to 19 COG functional categories.

**Fig 7:**
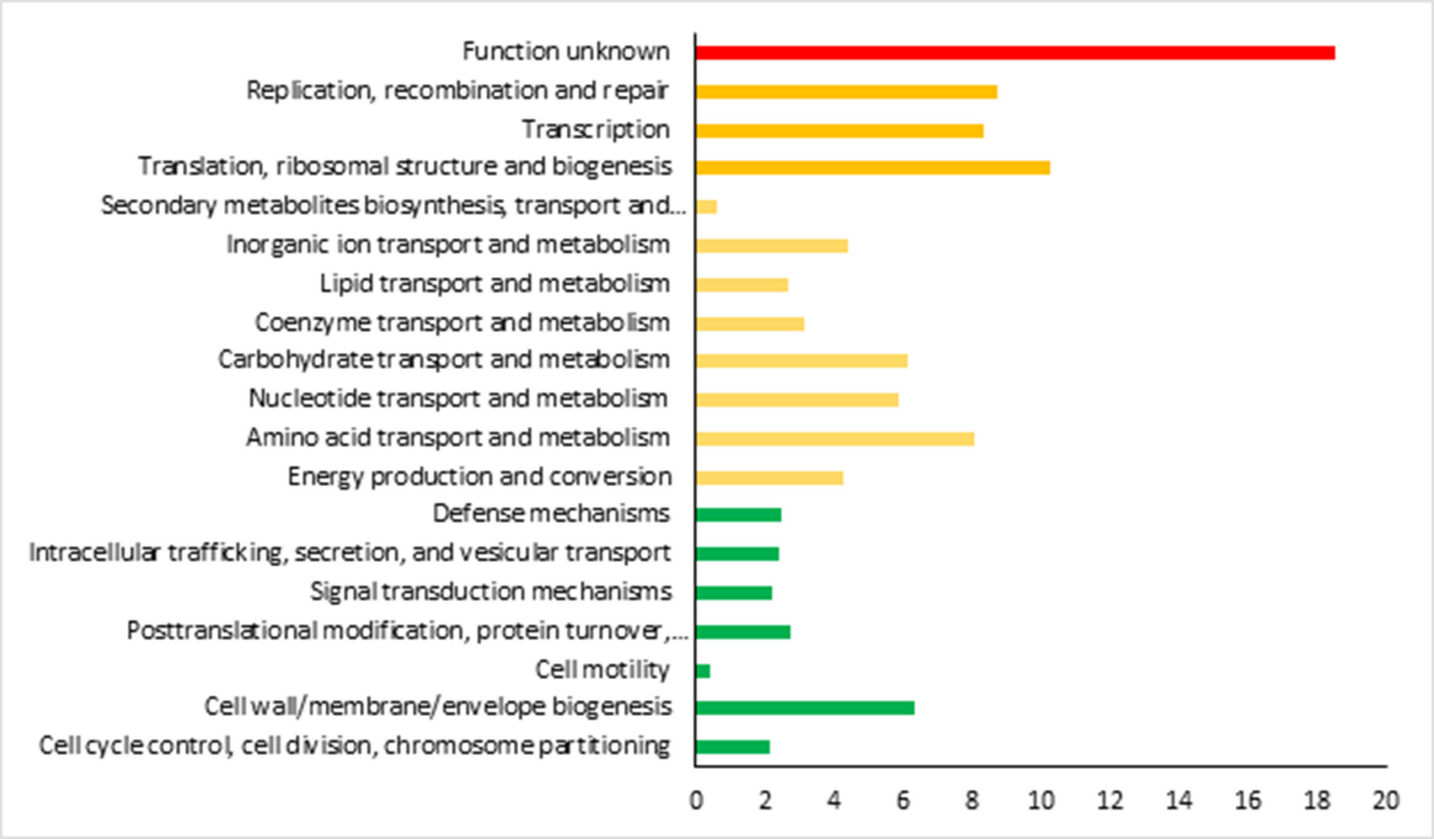
Classification of *L. salivarius* L33 total genes assigned to COG categories using eggNOG-mapper.

The majority of proteins were categorized based on their identified functions (81%), with subdivisions into translation, ribosomal structure, and biogenesis (10.2%), replication, recombination, and repair (8.7%), transcription (8.3%), among others (Fig. 7). To further enhance functional characterization, an analysis of the KEGG profile of *L. salivarius* was conducted. Approximately 56.9% of genes were assigned to 32 functional groups and 145 pathways. The predominant encoded proteins were found to participate in pathways related to carbohydrate metabolism, amino acid metabolism, and transcription.

Additionally, a CAZyme analysis was conducted using the database (https://bcb.unl.edu/dbCAN2/index.php) to identify carbohydrate-active enzymes in *L. salivarius*. The findings revealed the presence of 39 genes categorized into 4 CAZyme gene groups: 10 glycoside hydrolase (GH) genes, 25 glycosyltransferase (GT) genes, 2 carbohydrate-binding modules, and 2 carbohydrate esterase (CE) genes.

### Evaluation of probiotic traits

Building upon previous studies that identified genes associated with potential probiotics in *L. salivarius* and other probiotic bacteria (30, 31), the genome of *L. salivarius* L33 was analyzed for the presence of genes encoding proteins with putative probiotic functionalities. These functionalities include stress resistance, DNA and protein protection/repair, adhesion capabilities, immunomodulation, and bile salt tolerance. A BLAST analysis identified 19 genes in *L. salivarius* L33 that potentially encode proteins associated with these probiotic properties (Table 8). Notably, the genome harbors the nhaC gene, which encodes a Na+/H+ antiporter potentially contributing to low pH tolerance. Additionally, genes encoding proteins involved in heat stress response, such as dnaj, dnak, and htpx, were identified. Furthermore, the presence of protease genes like clp suggests a role in stress response and protection against protein damage. The potential for *L. salivarius* L33 to withstand oxidative stress environments is suggested by the presence of genes encoding proteins like glutaredoxin and thiolperoxidase.

**TABLE 8:** Proteins encoded by *L*. *salivarius* genome involved in probiotic mechanisms

Protein	Uniport	Function		Ref.
clpp	Q1WTA8	DNA and protein protection and repair	Survive in harsh environments	(30, 32)
clpb	A0A1D7TSG7			
msrb	Q1WVT3		Protection of bacteria against oxidative damage	(33)
dnaj	A0A1D7TT76	Stress resistance genes	Temperature tolerance	(34)
dnak	Q1WUE8			
htpx	Q1WV87		Heat shock protein	(35)
nhac	A0A2G4RJE5		Acid resistance	(36)
dnak	Q1WUE8	Adhesion ability	Mucin binding	(37)
Eno	Q1WSY0		Collagen binding	
MucBP	Q1WUZ1		Mucus binding	(30)
GroEL			Cell adhesion	
Rqch	Q1WTS3		Fibronectin binding	
LPXTG			Cell wall adhesion	
gndA	A0A7 × 2MEU0	Bile salt tolerance		(38)
Eno	Q1WSY0			
bsh1	Q1WR93			(30)
GNAT family N-acetyltransferase	Q1WTM6			
trxA	Q1WS50	Immunomodulation	NADPH-depend oxidative stress resistance	(39)
trxB	C2EHJ5			

The RGI analysis revealed that the genome of *L. salivarius* does not contain transferable antibiotic resistance genes. However, examination of the genome using amrfinder revealed the presence of a gene encoding the tetracycline resistance ribosomal protection protein Tet (M) and the tetracycline efflux MFS transporter Tet (L). Further analysis with BAGEL4 demonstrated the presence of a gene responsible for producing salivaricin P, a bacteriocin.

## DISCUSSION

This study investigated the safety-related probiotic properties and the preliminary genome sequence of *Ligilactobacillus salivarius* L33, a strain isolated from Arian broiler chickens. Ensuring the safety of probiotic strains is a critical prerequisite for their application in food and feed. To evaluate its suitability as a probiotic, comprehensive safety assessments were conducted on *Ligilactobacillus salivarius* L33. The strain exhibited no hemolytic activity on blood agar, indicating it does not lyse red blood cells, a key safety criterion for probiotics. Antibiotic resistance is a critical factor in evaluating the safety of probiotic strains. In this study, antibiotic susceptibility testing following CLSI guidelines showed that *L. salivarius* L33 is susceptible to most clinically relevant antibiotics, with no evidence of acquired transferable resistance genes. However, this strain exhibited intermediate resistance to tetracycline, which is consistent with previous reports of intrinsic tetracycline resistance in *L. salivarius* and other lactic acid bacteria (LAB). Genomic analysis revealed the presence of tet (M) and tet (L) genes, which encode ribosomal protection proteins and efflux transporters, respectively. These genes are chromosomally encoded and were not associated with mobile genetic elements, suggesting that the resistance is intrinsic rather than acquired. Intrinsic resistance in LAB is a well-documented phenomenon and is not considered a safety risk, as it does not contribute to the horizontal transfer of antibiotic resistance genes (40, 41). Furthermore, *L. salivarius* L33 remained susceptible to other clinically important antibiotics, supporting its potential as a safe probiotic candidate. Future research may further investigate its antibiotic resistance profile in *in vivo* models to confirm its stability and safety. Furthermore, genomic screening confirmed the absence of known virulence factors, toxins, and mobile genetic elements associated with antibiotic resistance dissemination. These findings were aligned with the safety criteria established by EFSA and previous studies on *L. salivarius* strains used in probiotic applications (42, 43). Although *in vivo* toxicity or cytotoxicity studies were not conducted in this study, the results strongly support the safety profile of *L. salivarius* L33. Future research could further validate its safety through *in vivo* assessments in animal models to confirm its non-pathogenicity and long-term effects on host health.

Genomic analysis revealed that the total genomic size of *Lactobacillus salivarius* L33 is 1,925,881 bp, with a GC content of 32.62%. These genomic characteristics of *L. salivarius* L33 are comparable to those observed in other *L. salivarius* strains, including closely related strains such as *L. salivarius* IBB3154, DJ-sa-01, and SNK6. The study showed that the genes of *L. salivarius* L33 are involved in the complete biosynthesis of three amino acids (Table 9) and encode such proteins needed for the biosynthesis of the rest of the amino acids. Furthermore, L33 is able to synthesize such amino acids by interconversion. For example, this genome is able to interconvert Asp to Asn via aspartate-ammonia ligase encoded by asnA, Ser to Gly via serine hydroxymethyltransferase encoded by glyA, and gln to glu and vice versa via glutamine synthetase encoded by glnA. In summary, *L. salivarius* L33 can synthesize *de novo* or by interconversion six amino acids and is auxotrophic for 14 amino acids. This auxotrophic level is consistent with the presence of numerous transporters, including ATP-dependent ABC transporter, di- and oligo transporter system (opp A, B, D, and F), transporter recognizing glutamine, spermidine/putrescine transporter, and permease for amino acid. Due to the strain’s inability to synthesize amino acids, it relies more heavily on the uptake of extracellular amino acids and thrives in nutrient-rich environments. Various research studies have demonstrated that *L. salivarius* colonizes in the gastrointestinal tract of various organisms, including humans (44), murines (45), swine (46), and chickens (47).

**TABLE 9:** Amino acids produced in bacterial genome

Amino acid	Status	No. of genes in the genome	KEGG module
Thr	Complete	5 out of 5	M00018
Cys	Complete	2 out of 2	M00021
Pro	Complete	2 out of 2	M00015

Carbohydrate metabolism is an important trait supporting the probiotic potential of LAB (48). Due to the contribution of carbohydrate metabolism in cellular processes such as energy production and stress processes, this metabolism plays a crucial role in the fitness of *Lactobacillus* in the ecological niche. Enzymes involved in carbohydrate metabolism facilitate the breakdown of indigestible and complex oligosaccharides, enhancing the growth and survival of the bacterial species within the host organism (49). Our analysis of the genome of *L. salivarius* categorized 39 genes as CAZymes. The genome contains 10 genes that encode GH, 25 genes that encode GT, 2 genes that encode carbohydrate-binding modules, and 2 genes that encode CE. Genes encoding CAZymes are crucial for bacterial adaptation to the gastrointestinal environment, as they facilitate the provision of nutrients for the growth of gut microbiota (50). It has been shown that bacteria encoding CAZymes in poultry microbiota are able to break down plant-derived fibers and degrade dietary carbohydrates and host-derived glycans (51). Among the CAZymes characterized in the *L. salivarius* L33 genome, the most abundant category corresponds to GTs. This CAZyme catalyzes a glycosylation reaction and produces structures facilitating adhesion to host cell mucoproteins (52).

The COG server assigned the strain-specific genes of the strain into such categories, including replication, recombination, and repair, transcription, and defense mechanisms, with most of them involved in replication, recombination, and repair. The genes assigned in transcription control the expression of genes and confer an advantage when present in the host intestine. Here, a gene assigned to the transcription class encoding Cro/C1-type HTH DNA-binding domain was identified. This protein regulates bacteriocin production in probiotic bacteria (53, 54). Among the genes related to the defense mechanism, a gene encoding type I and II restriction modification system was identified. Active restriction modification system prevents exogenous DNA and horizontal gene transfer (55, 56).

Probiotic bacteria often encounter harsh environments in the gastrointestinal tract, such as high acidity and the presence of bile salts. The capability to endure stressful conditions is an important feature in probiotic bacteria. Our analyses revealed that the genome contains genes that encode proteins involved in acid and bile tolerance, antimicrobial activity, and resistance to temperature (Table 8). The NA^+^/H^+^ antiporter encoded by the Nhac gene improves the fitness of bacteria in low pH conditions. This cation/proton antiporter exchanges cytoplasmic Na^+^ or K^+^ for H^+^ from outside the cell and has been reported in probiotic bacteria (36). The Clp proteins were also detected in the *L. salivarius* L33 genome. This protein plays a crucial role in protein quality control under normal and stressful conditions. This molecular chaperone complex helps in the correct folding of proteins and the degradation of misfolded proteins (32, 57). The proteins responsible for enabling bacteria to interact with their surrounding environment, such as LPXTG, GroEL, DnaK, MucBP, EF-TU, and ENO, were identified within the genome. LPXTG is a multifunctional protein that contains a conserved LPxTG motif in its C-terminus. In addition to providing bacteria with adhesion properties, this protein also exhibits anti-inflammatory effects by inhibiting the expression of inflammatory cytokines (58, 59). The moonlight proteins, GroEL and EF-Tu, are highly conserved proteins that not only aid bacteria in adhering to epithelial cells and mucus but also promote cytokine secretion in macrophages and facilitate the aggregation of pathogenic bacteria (30, 60, 61). Soni et al. (62) also identified these prominent moonlight proteins in the genome of the probiotic bacterium *Bacillus velezensis* CGS1.1, which was isolated from a chicken fecal sample. *L. salivarius* L33 harbors a gene that encodes bacitracin salivaricin P. This bacitracin is a two-component bacteriocin that has been reported to be produced by various strains of *L. salivarius* (63, 64). The production of this bacteriocin is a common characteristic of *L. salivarius* strains originating from the intestine. Different types of salivaricin, including salivaricin P, T, L, and SMXD5, have been found in *L. salivarius* strains isolated from human, porcine, and poultry. Messaoudi et al. (65) purified salivaricin from *L. salivarius* SMXD51 and demonstrated its promising effect on inhibiting food-borne pathogens (*Campylobacter* and *Listeria monocytogenes*) in poultry. Our bioinformatics analyses demonstrated the resistance of the studied genome to the antibiotic tetracycline. However, the resistance against tetracycline and kanamycin is considered to be an intrinsic feature of *L. salivarius* (40). It should be noted that the European Food Safety Authority has granted and provided Qualified Presumption of Safety for using *L. salivarius* as food and feed additives (42).

### Conclusion

The findings suggest that the *Ligilactobacillus* strain in question exhibits promising characteristics that make it a potential probiotic. Specifically, the *L. salivarius* L33 strain appears to be a strong contender for creating new probiotic formulations or as an ingredient in synbiotics when paired with an appropriate prebiotic. The ability of the strain to withstand the gastrointestinal journey and establish itself in the intestines is attributed to its capacity to adhere to epithelial cells and mucus. Additionally, this strain has the potential to inhibit the colonization of harmful bacteria, thereby reducing the risk of infection transmission. Consequently, incorporating the probiotic *Ligilactobacillus* strain could enhance the safety and quality of meat and other food products sourced from poultry.

## Data Availability

The genome sequences analyzed in this study are publicly available in the NCBI database under the following accession numbers: GCF_000008925.1 (UCC118), GCF_000143435.1 (CECT 5713), GCF_000758365.1 (JCM1046), GCF_001011095.1 (Ren), GCF_001723525.1 (CICC 23174), GCF_002162055.1 (ZLS006), GCF_002735985.1 (BCRC 12574), GCF_002736025.1 (BCRC 14759), GCF_003316955.1 (DJ-sa-01), GCF_011045395.1 (IBB3154), GCF_013487885.1 (2D), GCF_014841055.1 (ZLp4b), GCF_020535185.1 (AR809), GCF_021266585.1 (BNS11), GCF_021432185.1 (2102-15), GCF_023573545.1 (S01), GCF_024397675.1 (SNK-6), GCF_024466835.1 (L.S.05), GCF_024637975.1 (VHProbi A17), GCF_024665615.1 (SS-258), GCF_029743055.1 (P1CEA3), GCF_029917105.1 (AR612), GCF_030062785.1 (S92), GCF_030062805.1 (S99), GCF_030062825.1 (S32), GCF_030062845.1 (S35), GCF_030062875.1 (S39), GCF_030062895.1 (S40), GCF_030062915.1 (S91), GCF_030062935.1 (S96), and GCF_030169145.1 (ZSA5). The pan-genome data generated and analyzed during this study are available from the corresponding author upon reasonable request. The sequencing data generated in this study have been deposited in the NCBI Sequence Read Archive (SRA) under BioProject accession PRJNA1233803, BioSample accession SAMN47278806, and SRA accession SRR32628241.
