# Erratum for Maniee et al., “Whole-genome analysis of *Ligilactobacillus salivarius* L33, a potential probiotic strain isolated from chicken gastrointestinal tract”

**DOI:** 10.1128/spectrum.03200-25

**Published:** 2025-12-08

**Authors:** Seyedeh Arezoo Maniee, Sahar Mahmoodian, Javad Zamani Amirzakaria, Amir Meimandipour, Vahid Shariati, Elahe Tavakol

## ERRATUM

Volume 13, no. 10, e01591-24, 2025, https://doi.org/10.1128/spectrum.01591-24. Affiliation 4 should appear as shown below:

Department of Plant Genetics and Production, Shiraz University, Shiraz, Iran

